# The Incompatibility of Chloral Hydrate in the Presence of Potassium Bromide and Alcohol

**Published:** 1885-10

**Authors:** 


### The Incompatibility of Chloral Hydrate in the Presence of Potassium Bromide and Alcohol.

Prof. Geo. F. H. Markoe, in experimenting with solutions of chloral hydrate, finds this drug incompatible with bromide of potash and alcohol. He says experiments prove that the alcohol is the cause of the trouble, and the writer thinks that the chloral hydrate is changed into the less soluble chloral alcoholate. The writer found that the addition of potassium bromide, sodium chloride, and magnesium sulphate, to strong solutions of chloral hydrate, together with the presence of alcohol, determined a separation of the liquids into two layers. Ammonium chloride, ammonium bromide, potassium nitrate, and calcium bromide, did not disturb the same chloral solutions.

The practical lesson to be learned from this incompatible prescription, is that the alcoholic preparations should not be prescribed with chloral hydrate, especially not in connection with the bromides of potassium and sodium, because, if concentrated solutions are used, the chloral will separate as analcoholate, float on the surface, and a great risk will be incurred of giving a large overdose, the patient having received no caution with regard to the necessity of shaking the contents of the bottle before taking a dose.—*Boston Medical and Surgical Journal.*

This extract will be found of great interest to the profession. For the first time, our attention is called to the incompatibility, of drugs that are prescribed almost daily; for, most of the physicians called upon to treat delirium tremens or nervous diseases in general, rely on these drugs. Personally, we have never used these drugs in combination, preferring to prescribe each in a separate solution; either drug can then be increased or diminished from hour to hour, as needed. In many of the cases where these remedies are exhibited, it is necessary todiscontinue the chloral and increase the bromides when a certain effect is produced by the action of the chloral. Then, too, we know that chloral is a depressant of the cardiac and respiratory systems; hence, when a certain effect is produced by chloral, we usually resort at once to stimulants, which is the great argument for not using them in combination. But, as will be seen from the above, we must proceed on quite another basis—that of incompatibility. This discovery may explain many of the accidents that physicians report from time to time, from even 5 to 10 grains of chloral.

Bartholow, in speaking of the incompatibles of chloral, says, "Alkalies decompose chloral with the production of formic acid and chloroform, hence all agents having an alkaline re-action are incompatible," but this does not, as we all know, cover this point which we have so recently had called to our notice.

There is another point in the use of chloral which has been accepted as conclusive by the experienced practitioners in this community. It has been taught for years by a well-qualified teacher, and it would seem his conclusion must be true. I mean the incompatibility of chloral and morphia. In certain diseases of the nervous system, these remedies, in combination, are very useful. It is necessary, of course, to proceed carefully in the exhibition of the combination; when this is done, we can look for no untoward result.

Bartholow says of the use of this combination: "Chloral is not unfrequently prescribed to relieve pain by suspending the functions of the cerebrum, and in doses, therefore, which are dangerous. It has no direct pain-relieving power like morphia. When pain is to be relieved and sleep procured, the combination of chloral and morphia is extremely effective." The combination is also recommended by Clouston in insanity, and many others. While we are on this subject, let me call your attention to the care which should be exercised in its administration to patients worn out with prolonged drink or excessive mental activity. "It is more particularly adapted to those cases in which the delirium has succeeded to a debauch," or when the mental activity is of a recent date, and the patient is not exhausted. The use of chloral is attended with glorious results, its abuse with most disastrous.

The following significant paragraph appears in the *Medical News*, of Philadelphia, and is another indication of the necessity of requiring a higher standard of preliminary qualification of students by medical schools and by physicians acting as preceptors: "The occurrence of a druggist's mistake in Hoboken, whereby two young women lost their lives through the substitution of the sulphate of morphine for quinine, has given rise to a great deal of newspaper discussion. Several druggists, who have been interviewed, call attention to the looseness of prescription writing and the tendency to abbreviations. Though it seems, upon the part of physicians, almost too much to expect an enforced classical course in these days, when men study medicine as they would plumbing, *it certainly should be required that they be made to acquire sufficient knowledge of Latin to order medicines.*"

---

*L'Union Medicale du Canada*, published in Montreal, complains of the inactivity of the Board of Health in that city, in the presence of the epidemic of small-pox. The quarantine hospital is overcrowded with patients, and no measures are taken to enlarge it; the disease is constantly spreading, and almost nothing is done to retard its progress. About the only preventive measures adopted are the disinfection of rooms after the recovery or death of patients, and placing the word "picotte" upon the doors of infected houses.

---

We call attention to advertisement of PEPTONIZED BEEF in this issue. It would appear that the problem of an extractive of digested beef has been solved by Prof. Preston B. Rose, formerly of the Michigan State University, and its preparation attempted upon a scale commensurate with its importance. The General Agents of this preparation, MESSRS. CHAPMAN, GREEN & CO., OF CHICAGO, will be pleased to forward samples as per their advertisement.

